# Access to Healthcare Services among Thai Immigrants in Japan: A Study of the Areas Surrounding Tokyo

**DOI:** 10.3390/ijerph20136290

**Published:** 2023-07-04

**Authors:** Sopak Supakul, Pichaya Jaroongjittanusonti, Prangkhwan Jiaranaisilawong, Romruedee Phisalaphong, Tetsuya Tanimoto, Akihiko Ozaki

**Affiliations:** 1 Graduate School of Medicine, Keio University, Tokyo 160-8582, Japan; 2 Medical Governance Research Institute, Tokyo 108-0074, Japan; 3 Graduate School of Medical and Dental Sciences, Tokyo Medical and Dental University, Tokyo 113-8510, Japan; 4 American School in Japan, Tokyo 182-0031, Japan; 5 Navitas Clinic, Tokyo 190-0012, Japan; 6 Department of Breast and Thyroid Surgery, Jyoban Hospital of Tokiwa Foundation, Fukushima 972-8322, Japan

**Keywords:** undocumented immigrants, socioeconomic status, health profile, universal healthcare coverage (UHC), Thailand, Japan

## Abstract

Numerous undocumented and uninsured foreigners living in Japan have faced barriers when trying to obtain appropriate healthcare services, which have occasionally led to issues with unpaid medical bills to medical institutions. Although information on health and socioeconomic status is essential to tackle such issues, relevant data has been unavailable due to difficulties in contacting this population. This study involved a cross-sectional survey using questionnaires concerning the general demographic characteristics, socioeconomic status, health profiles, information access, and knowledge/attitude/practice of health insurance of Thai nationals living in Japan. The study participants included Thai nationals who lived in Tokyo and the surrounding prefectures. The survey was conducted mainly at public religious events from September 2022 to December 2022. Overall, the questionnaires were obtained from 84 participants, though 67 participants were included in the final analysis after excluding missing variables. There were participants with unspecified visa status (32.8%) and uninsured status (40.3%). Among them, 86.4% expressed positive attitudes towards health insurance. However, multivariate multivariable regression analyses revealed the low insurance practice status among the unspecified visa group (aOR, 0.02; 95% CI, 0.00–0.13). Overall, the results reveal limited access to healthcare services in subgroups of Thai immigrants in Japan.

## 1. Introduction

The economic constraints in their own countries and the purpose of seeking better opportunities have often resulted in the relocation of people from one country to another, especially from developing and underdeveloped countries to developed countries [1]. The relocation of immigrants has both advantages and disadvantages. Even though it could lead to better opportunities and income, the risk of living in a poor environment, encountering language barriers, and not being able to receive proper healthcare services remain valid concerns [2]. Japan is among the countries that have started to accept more immigrants due to government policies designed to promote an economic boost in the 21st century. The number of foreigners residing in Japan has increased significantly over the last 20 years [3]. Among the foreigners residing in Japan, there are documented immigrants with permanent residency status as well as immigrants with visas for working and studying. On the other hand, there are also some undocumented immigrants who reside in the country illegally.

Currently, Sustainable Development Goals (SDGs) aim to ‘leave no one behind’, including immigrants who can be at higher risk for poor health problems [4]. Immigrants have been identified as a vulnerable population in accessing adequate healthcare services due to various factors, such as language barriers, socioeconomic status, and migration status [5]. Especially for undocumented migrants, the laws and policies limit their opportunity to achieve better health status, excluding them from the right to health. Also, the fear of deportation, whether real or imagined, is a barrier to healthcare assessment. This forces immigrants to avoid using healthcare services and wait until their illness becomes more serious and expensive to treat [6,7].

In Japan, foreign residents are obligated to enroll for public medical insurance, either the National Health Insurance (NHI) for those who are unemployed, work less than 30 h per week, and students, or Social Insurance (SI) for those who are employed full-time [8]. Additionally, there is private insurance, which is commonly used for those with long-term illnesses and travelers. However, some clinics and hospitals in Japan may not easily accept the international healthcare plan [8]. Therefore, several cases including undocumented immigrants, uninsured travelers, and some private health insurance holders may fall into the gap in healthcare access in the country [9]. Uninsured status can cause confusion and difficulties for healthcare workers when providing support [10]. Since the uninsured must cover all their own medical costs after receiving treatment, there is an increased risk of unpaid medical bills to medical institutions, which occasionally becomes a problem [10,11]. It is often complicated to support the uninsured when being admitted to a hospital because of financial and language barriers. For them, various stakeholders, including the immigrant’s family, hospital, and governmental agencies, are often involved [12]. Although it is well acknowledged that the vulnerable group of immigrants, including the uninsured, often encounter financial issues that lead to unpaid medical bills, the socioeconomic status and health profiles of the vulnerable population before being admitted to a hospital are still unknown.

Since 2022, the number of immigrants living in Japan has been close to 2.5 million, and this figure is on the rise [13]. However, little is known about accessibility to healthcare among immigrants in Japan. Within this group, more than 50,000 registered Thai nationals currently reside in Japan [14]. However, the Immigration Services Bureau of Japan revealed that there are more than 7000 Thai nationals illegally residing in Japan as of 2022 [15]. Compared to other nationals, the proportion of undocumented to documented Thai immigrants gradually increased to 15% in 2022, which was the second highest in the world following Malaysia. Therefore, focusing on Thai nationals can provide valuable insights into the factors affecting access to healthcare for immigrants. Thus, this study conducted a cross-sectional survey using a questionnaire to address this population. Altogether, this study aimed to (i) report descriptive information on the socioeconomic status and health status of the vulnerable group of Thai foreigners living in Japan and (ii) assess factors associated with health insurance, including knowledge/attitude/practice.

## 2. Materials and Methods

### 2.1. Study Setting, Sample Size, and Data Collection

**Figure 1:**
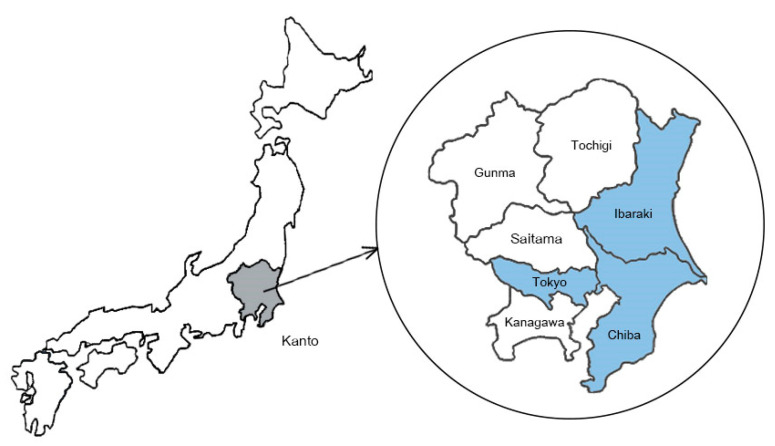
A survey was conducted in Tokyo and the surrounding Kanto area of Japan.

The questionnaires on socioeconomic and healthcare status were collected from Thai nationals aged 20 years or older residing in Tokyo and its surrounding prefectures. Data collection was mainly carried out at public religious events from September 2022 to December 2022. The sites where the survey was conducted were Ibaraki prefecture, Chiba prefecture, and Tokyo, which are in the Kanto area of Japan (Figure 1). For each event, there were more than 200 expected participants. In particular, there was a high opportunity for vulnerable groups of Thai nationals, including the uninsured, to participate in these events.

The questionnaires included items consisting of the general demographic characteristics, socioeconomic status, health profile, health information access, and knowledge/attitude/practice of health insurance among the participants. To avoid bias from the educational background of each participant, the researcher accompanied each participant when filling in the questionnaire and collected the questionnaire at the end. The estimated time to complete each questionnaire was 20 min.

### 2.2. Variable Measurements: Dependent Variables

The dependent variables included in this study consisted of catastrophic health expenditure (CHE), health insurance knowledge, attitude, and practice (KAP). CHE was calculated based on the annual household out-of-pocket (OOP) and annual income. The incidence of CHE was considered when there was an incidence of annual household OOP exceeding 40% of household annual income in the past [16,17]. The answers were either Yes or No. A “Yes” answer referred to the experience of having CHE incidence in the past without giving the specific time frame of the incident.

$$\mathrm{CHE}\;\mathrm{incidence}:\;\frac{\mathrm{Annual}\;\mathrm{household}\;\mathrm{OOP}}{\mathrm{Annual}\;\mathrm{household}\;\mathrm{income}}>40\%$$

Additionally, the health insurance knowledge/attitude/practice was assessed by asking the following questions: Do you know that there is a health insurance system in Japan? Do you know that health insurance can help reduce healthcare costs? Do you think that having health insurance is necessary? Do you have health insurance? 

Moreover, the health insurance knowledge was assessed by asking about the existence and benefits of having health insurance. The answer was bivariate as Yes or No. The results were interpreted as correct and incorrect knowledge. The health insurance attitude was examined through the perspective of the necessity of having health insurance. The results were interpreted as a positive attitude and a negative attitude. Finally, the health insurance practice was assessed by asking whether participants had any insurance. The results were interpreted as favoring or not favoring the practice. In addition, barriers to having healthcare insurance (having no visa, financial barrier, language barrier, complicated procedure, time constraint, insurance is not necessary, none) were assessed to understand the possible factors associated with the practice of having health insurance.

### 2.3. Variable Measurements: Independent Variables

The independent variables included demographics, socioeconomic status, Japanese language proficiency levels, health profiles, and health information access. The demographic variables were comprised of gender (male, female, other), age, education (elementary school or lower, junior high school, high school, bachelor’s degree or higher), registered hometown (central, eastern, northeastern, northern, and southern regions of Thailand), duration of stay in Japan (less than 6 months, 6 months to 5 years, more than 5 years), and visa type (short-term (less than 90 days—student, tourist, family visiting, and temporary visa), long-term (student, working, high skill professional, and marriage visa), and unspecified). Furthermore, the socioeconomic status was comprised of employment status (full-time employed, unemployed, own business, part-time, and other), annual household income, and annual household out-of-pocket (OOP) healthcare expenditure. The Japanese language level (Japanese Language Proficiency Test (JLPT) N5, N4, N3, N2, N1, and lower than N5) was used for the evaluation of language skills while residing in Japan.

The health profile included the data collection of insurance type (National Health Insurance (NHI), Social Insurance (SI), other, none) and diagnosed chronic diseases (hypertension, diabetes mellitus (DM), dyslipidemia, cardiovascular diseases, thyroid disease, mental health disease, cancer, other, none). Additionally, the health information access variables included language used when receiving healthcare services (Thai, Japanese, English, other), source of healthcare information social network services (SNS), which represent the governmental sources, and non-governmental SNS sources (television/radio/newspaper, friends/family members, employers, other). Furthermore, the variable for unspecified type of visa, which referred to those who had no visa or were illegal immigrants, was used as the independent variable and was analyzed separately in descriptive statistics.

### 2.4. Analysis

SPSS version 29.0 statistical software (IBM SPSS, Armonk, NY, USA) was used for data analysis. The descriptive statistical analyses were implemented to describe demographic characteristics and socioeconomic status, including CHE incidence, health profile information, health information access, and knowledge/attitude/practice of health insurance of the study population by showing percentage and frequency. Subanalysis among those who had “Unspecified visa status” was analyzed in descriptive statistics. The association between CHE, knowledge, attitude, and practice of having health insurance and sociodemographic–socioeconomic status (SES) were analyzed and interpreted by chi-square. Multivariate regression analysis was also employed to assess the association of CHE and KAP to demographic characteristics and SES. In particular, CHE incidence and health insurance knowledge, attitude, and practice were set as dependent variables, while educational level, duration of stay, and type of visa were set as independent variables. The independent variables included in the multivariate multivariable model were selected from the variables among other demographic characteristics and SES that showed a significant correlation with the dependent variables from the chi-square test. The significance was set at *p* < 0.05. The adjusted odds ratio (aOR) and 95% CI were calculated to examine the associations between variables.

### 2.5. Ethics and Consent

This study was approved by the Institutional Review Board of the Medical Governance Research Institute, Tokyo, Japan (certificate no. MG2020-10-R1). All participants agreed to provide informed consent before the initiation of the study.

## 3. Results

The total number of participants collected in this survey was 84. However, a total of 67 participants (79.8%) were included in the final analysis after excluding those participants who had missing data.

Among the 67 participants, 43 (64.2%) were female and 24 (35.8%) were male. The educational levels varied from elementary school or lower to a bachelor’s degree or higher. The data on the registered hometown showed that most of the participants were originally from the northeast region of Thailand (43.3%), followed by the northern region (17.9%). Most of the participants stayed in Japan longer than 5 years (55.2%), while 10.4% stayed in Japan for less than 6 months. In addition, only a few participants held a Japanese language proficiency higher than the JLPT N5 level (4.5%). Regarding the visa type in this study population, there were 4 (6%) short-term visa holders, 41 (61.2%) long-term visa holders, and 22 (32.8%) unspecified visa holders (Table 1).

Among the study population, 59 participants were employees, and 73.1% were employed full-time. The household income also varied from less than 1,000,000 yen per year (10.4%) to more than 10,000,000 yen per year (1.5%). Most of the study participants reported having an annual income in the range of 2,000,000 yen to 2,999,999 yen. Moreover, most of the participants had out-of-pocket (OOP) healthcare expenditures of less than 400,000 yen a year (64.2%), while 1.5% reported having an OOP of more than 4,000,000 yen a year. Regarding the catastrophic healthcare expenditure (CHE) incidence, 63 (94%) had never had CHE incidence and 4 (6%) had CHE incidence in the past (Table 2).

Regarding the health insurance status of the participants, 25 (37.3%) and 13 (19.4%) were enrolled in National Health Insurance (NHI) and Social Insurance (SI), respectively. Interestingly, 40.3% reported having no insurance. For the health status, 41.8% were diagnosed with diseases. For health information access in this population, 79.1% needed to communicate with healthcare providers in Japanese when receiving healthcare services. The source of information on healthcare among the study population mostly came from friends and family (40.3%) and non-governmental social network services (SNS) sources (35.8%).

Among the study population, 82.1% knew that health insurance was provided in Japan and that it could help reduce the cost of medication. Furthermore, 94% thought that it was necessary to have insurance. Despite the positive attitude towards having health insurance, barriers to the practice of having insurance were comprised of language (29.9%), financial (23.9%), complicated procedures (7.5%), and time constraints (1.5%).

The information on unspecified visa groups was analyzed separately using descriptive results, as shown in Table 1, Table 2 and Table 3. Among these, 59.1% were aged below 30 years old, and 95.5% were aged below 50 years old. In addition, half of the unspecified visa-holder immigrants reported having high school educational levels (45.5%). However, they showed low proficiency in the Japanese language (77.2% were N5 and below). According to the socioeconomic status, 95.5% reported being employed, 68.2% had a household income of 2–3.9 million yen per year, and 90.9% had no CHE incidence.

The health insurance knowledge/attitude/practice (KAP) was also examined among the unspecified visa-holder group, with 90.9% of participants reporting having no health insurance and 81.8% possessing no underlying health condition. Japanese was the most common language used to receive sources of information (72.7%), while employers, family, and friends were chosen to be reliable sources of information (45.5% and 45.5%, respectively). Additionally, 63.4% of individuals had correct knowledge of health insurance and 86.4% had positive attitudes toward having health insurance. Although language and financial barriers were reported as common factors preventing participants from having health insurance (54.5% and 40.9%, respectively), having no residence status should be considered an absolute factor.

Bivariate analysis (chi-square) and multivariate multivariable regression analysis were performed to assess the association of the socio-demographic variables to CHE and health insurance knowledge/attitude/practice variables, as shown in Table 4. There was no association between the unspecified visa status and CHE incidence (aOR, 1.24; 95% CI, 0.13–11.52). The study population with an education level higher than high school had a significantly higher knowledge of health insurance (aOR, 11.85; 95% CI, 1.87–75.23) as well as those with a duration of stay longer than 5 years (aOR, 16.85; 95% CI, 1.61–176.62). Moreover, the unspecified visa population had a lower practice in health insurance (aOR, 0.02; 95% CI, 0.00–0.13).

## 4. Discussion

Despite having a relatively small population size, the current study is among the few that have revealed demographic characteristics for the socioeconomic status and health profiles of Thai nationals residing in Japan, especially those without legal residency status. Around one-third of participants were unspecified visa holders, which refers to those who had no visa or were overstayed immigrants. However, the proportion of catastrophic health expenditure (CHE) was fortunately low. Nonetheless, this fact should be cautiously interpreted because the majority of the unspecified visa status group were young adults with no underlying diseases. Since most undocumented immigrants work as laborers, they tend to be young and healthy, resulting in underutilization of health services [18]. However, they may be exposed to hazardous working conditions that could affect their occupational health and safety. Additionally, almost all undocumented immigrants lack health insurance, which can limit their access to healthcare services and increase their health risks in the future.

In this study, 6% had experienced CHE in the past. Similarly, a previous study on immigrant workers in Western China found a CHE incidence rate of 12.5% [19]. Another study conducted in China reported much higher rates for CHE (61.8% and 51.9%) among migrants in rural and urban areas [20]. However, this study defined CHE as out-of-pocket health expenditures in excess of 10% of annual family income, whereas our study used a 40% cut-off threshold. As such, it is difficult to make direct comparisons between these studies. Nonetheless, for the immigrants in Japan, there has never been any survey conducted to assess the CHE incidence. Since there is the possibility of CHE incidence among immigrants in Japan, preventive measures for the risk population are strongly encouraged, such as for those with low incomes [21].

This study found a low CHE incidence among the unspecified visa holders (9.1%) in our study compared to the incidence of CHE in Japanese households in general (CHE incidence rate of 8–12.4% at the 10% threshold and 1.1–2.2% at the 25% threshold during 2004–2020) [22]. This observation is possibly due to the young age of the cohort (20–29 years old (59.1%)) and no reported chronic diseases (81.8%). One study on undocumented immigrants also revealed that most of the cases with no insurance access to healthcare services are mainly due to injury and dental diseases, not chronic diseases [23]. Furthermore, the tendency of low-level Japanese language proficiency in this population might also contribute to language barriers to receiving healthcare services when needed. Despite the increasing prevalence of international migration, data on CHE among immigrants is still limited. Further research is needed for a better understanding of the health expenditure burden faced by immigrants and to form policies that can help to mitigate this burden.

The majority of participants could understand only some basic Japanese language (JLPT >/= N5). Communication ability is one of the major challenges for immigrants trying to access healthcare services [6], and only 4.5% in this study could communicate in a variety of circumstances (N1), while 16.4% could not understand any Japanese. Moreover, the main sources of healthcare information among the immigrants in this study were friends and family members (40.3%). The immigrants’ poor proficiencies in the host country’s language can restrict their assessment of health services [24]. Language barriers can also create a gap between healthcare providers and patients, leading to potential misunderstandings or lack of comprehension [25]. Services on providing healthcare information in a native language for the immigrants might help alleviate the health information access among the immigrants.

The current study indicated that Thai immigrants living in Japan tended to have high knowledge and positive attitude toward having health insurance; however, almost half of them reported holding no health insurance. The high proportion of immigrants without health insurance could conceivably be explained by financial and language barriers. These facts could be supported by various studies. For example, one study in Ghana suggested that immigrants, regardless of their residence status, often decided not to apply for health insurance due to the unpredictable out-of-pocket (OOP) expenses and the need to be absent from work [26]. Language and cultural gaps also created difficulties for immigrants in accessing healthcare and registering for the health system [27]. Even though higher education levels tended to increase the odds ratio of having better health insurance knowledge, a previous study found that awareness, rather than adequate knowledge, was more likely to influence immigrants to have health insurance [28].

The population with unspecified visa status in this study was less likely to show the practice of having health insurance. This is possibly explained by their illegal status prohibiting them from registering with the system. Despite the high considerable proportion of positive attitude towards health insurance (86.4%), having no status of residence prevents this population from receiving coverage from health insurance. Although attempts to include this population group in the national healthcare system are challenging for many countries, having healthcare insurance promotes healthcare accessibility and healthcare system communication among immigrants, which are necessary for preventing the spread of some serious diseases, including HIV [29]. One study showed a high prevalence of immigrants without public health insurance who were advised to seek medical care after consulting with doctors [30]. Thus, these observations indicated lower healthcare profiles and fewer preventive measures for immigrants, especially the vulnerable ones.

The government of Japan has declared a commitment to the Sustainable Development Goals (SDGs), especially to ensure healthcare services for everyone, and to achieve health for all by 2030. This includes children, persons with disabilities, people living with HIV/AIDS, older persons, Indigenous Peoples, internally displaced persons, refugees, and migrants [4,31]. The Tokyo Declaration on Universal Health Coverage in 2017 also clearly stated that Japan will accelerate progress towards UHC, thus achieving health for all people, whoever they are and wherever they live, by 2030 [32]. However, currently, there is no existing or developing scheme to protect the health status of undocumented immigrants. Some policies can be applicable to undocumented immigrants in a specific circumstance, such as the Public Expenditure System that covers the cost of medical treatment and follow-up of tuberculosis, regardless of the residence status of the patients [33].

In European countries, the UHC applies to undocumented immigrants. The levels of inclusiveness range from (i) access to only emergency services to (ii) greater access to some services to (iii) full access under specified conditions [34]. In the United States, hospitals must treat emergency patients regardless of their status, including undocumented immigrants, under the Emergency Medical Treatment and Labor Act (EMTALA) [35]. Despite being a developing country, Thailand has made the UHC available for everyone, its citizens and undocumented immigrants alike [36]. The changes in policies concerning access to healthcare services for asylum seekers and refugees in Germany between 1994 and 2013 have also concluded that immigrant-inclusive health systems would reduce healthcare expenditures in the long term [36,37].

In Japan, foreigners are required to have legal residence status to receive standard welfare services, which include enrolling in the health insurance system [38]. Japan is one of the most well-known countries to have achieved universal health coverage (UHC) for its citizens and even allows registered foreign residents who stay for 90 days or longer to enroll in their national health insurance system [38]. However, undocumented immigrants are a vulnerable population that is excluded from having healthcare insurance; if they receive healthcare services, they have to cover the full cost. This is the case even in life-threatening conditions [39]. Even though this study revealed the low rate of CHE incidence among these populations before hospital admission, CHE incidence is likely to occur when the individuals need hospitalization. Without an established supporting system like in other developed countries, this financial issue often causes concern and confusion for healthcare providers and sometimes results in high-cost unpaid medical bill issues [12,40].

Due to the decreasing birthrate, increasing population of the elderly, and stagnating economics in Japan, establishing a supporting scheme for these vulnerable people remains challenging [41]. Thus, retaining healthcare support for undocumented immigrants in Japan remains an unresolved problem at present. For some residents of foreign nationalities living in Japan, the embassies have played an important role in connecting a hospital and the family of the immigrant once admitted to the hospital, although it is often the case that support for medical fees is limited and only applies to certain conditions [12]. In terms of approaches to the immigrants and implementing health insurance for them, the illegal workers should be able to be legally registered with the healthcare system, specifically for funding for immigrants once admitted to the hospital without deportation back to their countries of origin [42,43].

There were some limitations in this study. First, the difficulty in contacting the population without residency status may have affected the sample size. Unless hospital admission is needed, immigrants in the population without a visa often disguise themselves for fear of being caught and deported from the country of residence. Therefore, we could only approach some of them who participated in the gathering of the Thai community in Japan. Due to the limited number of participants in this study and since more samples from other regions are still needed, the results might not represent the entire population of Thai immigrants residing in Japan and their relevant issues. Readers should interpret the results and the implications of this study with caution. Second, since this study used convenience sampling, it was difficult to recruit a very specific subgroup of immigrants. Those who were experiencing CHE incidences and had low health profiles might not have been present at the events where the survey was carried out. Using other methods is recommended in future studies, such as respondent-driven sampling, to recruit hard-to-reach populations [44]. Third, the CHE in this study was adopted and designed for the questionnaire utilizing the definition of CHE incidence as expenditure on healthcare exceeding 40% of the household income, according to the WHO [16]. Therefore, a lower percentage (10% and 25%) of healthcare expenditures on household income might result in an increase in CHE incidence in this study. The accuracy of estimating CHE incidence in our study could also be affected by the relatively small sample size. Lastly, the current study focused mainly on cases of Thai immigrants residing in Japan, but a comparison between the Thai population living in Japan and the Japanese population would provide a clearer understanding of the differences in accessing healthcare services in Japan.

## 5. Conclusions

The vulnerable group of Thai foreigners in Japan, especially those without legal residency status, had a low level of practice in terms of having insurance, despite possessing a high level of knowledge and positive attitude towards health insurance. Despite the limitation in the sampling approach in this study, some participants experienced incidences of CHE. Lack of residency status would prevent these populations from being covered by the country’s healthcare system and preclude them from receiving appropriate healthcare services. Even some documented immigrants were found to be uninsured. To ensure healthcare coverage for foreigners living in Japan, the development of an immigrant-inclusive healthcare system is needed. Consequently, identifying those with a high risk of CHE incidences should be carried out further to enable the provision of support.

## Figures and Tables

**Table 1:** General demographic characteristics of the study population (*n* = 67).

		Total Population (*n* = 67)		Unspecified Visa Status (*n* = 22)	
Variables		*n*	%	*n*	%
Gender					
	Female	43	64.2	10	45.5
	Male	24	35.8	12	54.5
Age (years)					
	20–29	18	26.9	13	59.1
	30–39	8	11.9	4	18.2
	40–49	15	22.4	4	18.2
	50–59	19	28.4	1	4.5
	≥60	7	10.4	0	0
Educational level					
	Elementary school or lower	18	26.9	2	9.1
	Junior high school	15	22.4	7	31.8
	Highschool	20	29.9	10	45.5
	Bachelor’s degree or upper	14	20.9	3	13.6
Hometown					
	Northeast	29	43.3	2	9.1
	Central	16	23.9	5	22.7
	East	7	10.4	11	50
	North	12	17.9	4	18.2
	South	3	4.5	0	0
Duration of stay					
	<6 months	7	10.4	5	22.7
	6 months–5 years	23	34.3	12	54.5
	≥5 years	37	55.2	5	22.7
Japanese language proficiency					
	less than N5	11	16.4	5	22.7
	N5	20	29.9	12	54.5
	N4	12	17.9	3	13.6
	N3	16	23.9	2	9.1
	N2	5	7.5	0	0
	N1	3	4.5	0	0
Type of visa					
	Short term	4	6		
	Long term	41	61.2		
	Unspecified	22	32.8		

Japanese proficiency level refers to the Japanese Language Proficiency Test (JLPT), where the levels were classified as N1–N5 according to the following criteria. N1: The ability to understand Japanese used in a variety of circumstances; N2: the ability to understand Japanese used in everyday situations and in a variety of circumstances to a certain degree; N3: the ability to understand Japanese used in everyday situations to a certain degree; N4: the ability to understand basic Japanese; N5: the ability to understand some basic Japanese.

**Table 2:** Socioeconomic status of the study population.

		Total Population (*n* = 67)		Unspecified Visa Status (*n* = 22)	
Variables		*n*	%	*n*	%
Employment status					
	Full-time employed	49	73.1	21	95.5
	Unemployed	8	11.9	1	4.5
	Business owner	5	7.5	0	0
	Part-time employed	1	1.5	0	0
	Other	4	6	0	0
Household income (100,000 Japanese yen/year)					
	<19	15	22.4	3	13.6
	20–39	33	49.3	15	68.2
	40–59	11	16.4	3	13.6
	60–79	2	3	0	0
	≥80	6	8.9	1	4.5
Out-of-pocket (OOP) (100,000 Japanese yen/year)					
	<4	43	64.2	11	50
	4.1–8	15	22.4	7	31.8
	8–12	6	9	3	13.6
	12.1–16	2	3	0	0
	40	1	1.5	1	4.5
Catastrophic healthcare expenditure (CHE)					
	No CHE	63	94	20	90.9
	Have CHE	4	6	2	9.1

**Table 3:** Health insurance status, health profile, health information access, knowledge, and attitude to health insurance of the study population.

	Total Population (*n* = 67)		Unspecified Visa Status (*n* = 22)	
Variables	*n*	%	*n*	%
Insurance type				
National Health Insurance (NHI)	25	37.3	0	0
Social Insurance (SI)	13	19.4	0	0
Other	2	3	2	9.1
None	27	40.3	20	90.9
Health status				
Chronic diseases	28	41.8	4	18.2
No chronic diseases	39	58.2	18	81.8
Health information access				
*Language used when receiving healthcare services*				
Thai	6	9	4	18.2
Japanese	53	79.1	16	72.7
English	5	7.5	1	4.5
Other	3	4.5	1	4.5
*Source of information*				
Non-governmental SNS sources	24	35.8	7	31.8
Governmental SNS sources	8	11.9	0	0
Television/radio/newspaper	7	10.4	0	0
Friends/family members	27	40.3	10	45.5
Employers	15	22.4	10	45.5
Other	7	10.4	1	4.5
Health insurance knowledge				
*Do you know that health insurance can help*				
*reduce the healthcare costs?*				
Yes	55	82.1	14	63.6
No	12	17.9	8	36.4
Health insurance attitude				
*Do you think that having health insurance is necessary?*				
Necessary	63	94	19	86.4
Not necessary	4	6	3	13.6
Health insurance barriers				
Language	20	29.9	12	54.5
Financial	16	23.9	9	40.9
Complicated procedure	5	7.5	0	0
Time constraint	1	1.5	2	9.1
Not necessary	3	4.5	1	4.5
No visa	6	9	4	18.2
No barrier	33	49.3	0	0

SNS: Social Network Services.

**Table 4:** Multivariate regression analysis of socio-demographic variables to CHE and health insurance knowledge/attitude/practice variables.

Variable	CHE				Knowledge HI				Attitude HI				Practice HI			
	aOR	95% CI	*p*-Value	X^2^ Test *p*-Value	aOR	95% CI	*p*-Value	X^2^ Test *p*-Value	aOR	95% CI	*p*-Value	X^2^ Test *p*-Value	aOR	95% CI	*p*-Value	X^2^ Test *p*-Value
Educational level																
Junior high school or lower																
High school or higher	0.98	0.13–7.60	0.984	0.975	11.85	1.87–75.23	0.009	0.014	0.45	0.04–5.44	0.533	0.317	1.366	0.27–6.99	0.708	0.686
Duration of stay																
<5 years																
≥5 years	0.28	0.02–3.44	0.316	0.21	16.85	1.61–176.62	0.025	<0.001	0.54	0.05–5.93	0.615	0.828	5.72	1.20–27.27	0.029	<0.001
Visa																
Have a visa																
Unspecified	1.24	0.13–11.52	0.851	0.451	0.59	0.02–1.08	0.059	<0.001	0.12	0.01–1.64	0.112	0.064	0.02	0.00–0.13	<0.001	<0.001

Values are presented as aOR with 95% CI. The aORs were adjusted by educational level, duration of stay, and type of visa. CHE = Catastrophic health expenditure; HI = health insurance; aOR = adjusted odds ratio; CI = confidence interval; Ref = reference.

## Data Availability

The data that support the findings of this study are available on request from the corresponding author. The data are not publicly available due to privacy or ethical restrictions.
